# Supplementary material for: Fibrillarin-mediated ribosomal RNA maturation is a novel therapeutic vulnerability in triple-negative breast cancer
Source: Breast Cancer Res. 2025 Nov 13;27:202. doi: 10.1186/s13058-025-02163-x (PMC12616997; doi:10.1186/s13058-025-02163-x)
Supplement: Supplementary file 1 — Supplementary Material 1 [file 13058_2025_2163_MOESM1_ESM.docx]

**Fibrillarin-mediated ribosomal RNA maturation is a novel therapeutic vulnerability in triple-negative breast cancer**

Camille Jouines^1*^, Piero Lo Monaco^1*^, Angéline Gaucherot^1*^, Julie Radermecker^1^, Caroline Isaac^1^, Fleur Bourdelais^1^, Marie-Ambre Monet^1^, Marion Meyer^1^, Mounira Chalabi-Dchar^1^, Flora Nguyen Van Long^1^, Laury Baillon^1^, Carine Froment, Julien Marcoux, Christophe Vanbelle^4^, Tanguy Fenouil^1^, Sébastien Durand^1^, Stéphane Giraud^5^, Jean-Jacques Diaz^1^, Virginie Marcel^1^, and Frédéric Catez^1^

**Supplementary tables S1 to S5**

Note: Supplementary tables S6 and S7 are provided separately as excel files.

**Supplementary Table S1**

"Ribosome biogenesis in eukaryotes" gene signature, defined by the KEGG pathway.

**Symbol Name**

AATF apoptosis antagonizing transcription factor

ABCE1 ATP-binding cassette, sub-family E (OABP), member 1

ABCF2 ATP-binding cassette, sub-family F (GCN20), member 2

ABT1 activator of basal transcription 1

ANKZF1 ankyrin repeat and zinc finger domain containing 1

DDX47 DEAD (Asp-Glu-Ala-Asp) box polypeptide 47

ATP8A1 ATPase, aminophospholipid transporter (APLT), class I, type 8A, member 1

ATP8A2 ATPase, aminophospholipid transporter, class I, type 8A, member 2

BCCIP BRCA2 and CDKN1A interacting protein

BMS1 BMS1 homolog, ribosome assembly protein (yeast)

BRIX1 BRX1, biogenesis of ribosomes, homolog (S. cerevisiae)

BYSL bystin-like

C12orf45 chromosome 12 open reading frame 45

C14orf169 chromosome 14 open reading frame 169

C1orf109 chromosome 1 open reading frame 109

EMG1 EMG1 nucleolar protein homolog (S. cerevisiae)

CASC1 cancer susceptibility candidate 1

EIF4A1 eukaryotic translation initiation factor 4A1

CEBPZ CCAAT/enhancer binding protein (C/EBP), zeta

IPO4 importin 4

CINP cyclin-dependent kinase 2 interacting protein

CIRH1A cirrhosis, autosomal recessive 1A (cirhin)

CMSS1 cms1 ribosomal small subunit homolog

COIL coilin

CRBN cereblon

CSNK2A2 casein kinase 2, alpha prime polypeptide

CSNK2B casein kinase 2, beta polypeptide

DBR1 debranching enzyme homolog 1 (S. cerevisiae)

DCAF13 DDB1 and CUL4 associated factor 13

DDX10 DEAD (Asp-Glu-Ala-Asp) box polypeptide 10

DDX18 DEAD (Asp-Glu-Ala-Asp) box polypeptide 18

DDX21 DEAD (Asp-Glu-Ala-Asp) box helicase 21

DDX24 DEAD (Asp-Glu-Ala-Asp) box polypeptide 24

DDX27 DEAD (Asp-Glu-Ala-Asp) box polypeptide 27

DDX28 DEAD (Asp-Glu-Ala-Asp) box polypeptide 28

DDX31 DEAD (Asp-Glu-Ala-Asp) box polypeptide 31

DDX49 DEAD (Asp-Glu-Ala-Asp) box polypeptide 49

DDX5 DEAD (Asp-Glu-Ala-Asp) box helicase 5

DDX51 DEAD (Asp-Glu-Ala-Asp) box polypeptide 51

DDX52 DEAD (Asp-Glu-Ala-Asp) box polypeptide 52

DDX54 DEAD (Asp-Glu-Ala-Asp) box polypeptide 54

DDX55 DEAD (Asp-Glu-Ala-Asp) box polypeptide 55

DDX56 DEAD (Asp-Glu-Ala-Asp) box helicase 56

DHX15 DEAH (Asp-Glu-Ala-His) box polypeptide 15

DHX33 DEAH (Asp-Glu-Ala-His) box polypeptide 33

DHX37 DEAH (Asp-Glu-Ala-His) box polypeptide 37

DIEXF digestive organ expansion factor homolog (zebrafish)

DIP2A DIP2 disco-interacting protein 2 homolog A

DKC1 dyskeratosis congenita 1, dyskerin

DNAJC21 DnaJ (Hsp40) homolog, subfamily C, member 21

DROSHA drosha, ribonuclease type III

DUSP12 dual specificity phosphatase 12

EBNA1BP2 EBNA1 binding protein 2

EFTUD1 elongation factor Tu GTP binding domain containing 1

EIF3J eukaryotic translation initiation factor 3, subunit J

EIF4A3 eukaryotic translation initiation factor 4A3

EIF6 eukaryotic translation initiation factor 6

ERAL1 Era G-protein-like 1 (E. coli)

EXOSC10 exosome component 10

FBL fibrillarin

FCF1 FCF1 small subunit (SSU) processome component homolog (S. cerevisiae)

FPR3 formyl peptide receptor 3

FTSJ2 FtsJ RNA methyltransferase homolog 2 (E. coli)

GAR1 GAR1 ribonucleoprotein homolog (yeast)

GNL2 guanine nucleotide binding protein-like 2 (nucleolar)

GNL3 guanine nucleotide binding protein-like 3 (nucleolar)

GRWD1 glutamate-rich WD repeat containing 1

GTPBP4 GTP binding protein 4

GTPBP5 GTP binding protein 5 (putative)

HEATR1 HEAT repeat containing 1

HSP90AA1 heat shock protein 90kDa alpha (cytosolic), class A member 1

HSPA1A heat shock 70kDa protein 1A

HSPA2 heat shock 70kDa protein 2

HSPA6 heat shock 70kDa protein 6 (HSP70B')

HSPA8 heat shock 70kDa protein 8

IGHMBP2 immunoglobulin mu binding protein 2

IMP3 IMP3, U3 small nucleolar ribonucleoprotein, homolog (yeast)

IMP4 IMP4, U3 small nucleolar ribonucleoprotein, homolog (yeast)

IPO5 importin 5

IPO7 importin 7

IPO9 importin 9

ISG20 interferon stimulated exonuclease gene 20kDa

ISG20L2 interferon stimulated exonuclease gene 20kDa-like 2

KDM8 lysine (K)-specific demethylase 8

KIAA0020 KIAA0020

KPNB1 karyopherin (importin) beta 1

KRI1 KRI1 homolog (S. cerevisiae)

KRR1 KRR1, small subunit (SSU) processome component, homolog (yeast)

LSG1 large subunit GTPase 1 homolog (S. cerevisiae)

LTN1 listerin E3 ubiquitin protein ligase 1

LTV1 LTV1 homolog (S. cerevisiae)

MAK16 MAK16 homolog (S. cerevisiae)

MDN1 MDN1, midasin homolog (yeast)

METTL18 methyltransferase like 18

METTL5 methyltransferase like 5

MINA MYC induced nuclear antigen

MPHOSPH10 M-phase phosphoprotein 10 (U3 small nucleolar ribonucleoprotein)

MRM1 mitochondrial rRNA methyltransferase 1 homolog (S. cerevisiae)

MRTO4 mRNA turnover 4 homolog (S. cerevisiae)

MTG1 mitochondrial GTPase 1 homolog (S. cerevisiae)

NAF1 nuclear assembly factor 1 homolog (S. cerevisiae)

NAT10 N-acetyltransferase 10 (GCN5-related)

NCL nucleolin

NEMF nuclear export mediator factor

NGDN neuroguidin, EIF4E binding protein

NHP2 NHP2 ribonucleoprotein

NHP2L1 NHP2 non-histone chromosome protein 2-like 1 (S. cerevisiae)

NIP7 nuclear import 7 homolog (S. cerevisiae)

NMD3 NMD3 homolog (S. cerevisiae)

NOA1 nitric oxide associated 1

NOB1 NIN1/RPN12 binding protein 1 homolog (S. cerevisiae)

NOC4L nucleolar complex associated 4 homolog (S. cerevisiae)

NOL12 nucleolar protein 12

NOL6 nucleolar protein family 6 (RNA-associated)

NOL8 nucleolar protein 8

NOL9 nucleolar protein 9

NOLC1 nucleolar and coiled-body phosphoprotein 1

NOP10 NOP10 ribonucleoprotein

NOP14 NOP14 nucleolar protein

NOP16 NOP16 nucleolar protein

NOP2 NOP2 nucleolar protein

NOP56 NOP56 ribonucleoprotein

NOP58 NOP58 ribonucleoprotein

NOP9 NOP9 nucleolar protein

NPAS4 neuronal PAS domain protein 4

NPLOC4 nuclear protein localization 4 homolog (S. cerevisiae)

NSA2 NSA2 ribosome biogenesis homolog (S. cerevisiae)

NSUN4 NOP2/Sun domain family, member 4

NSUN5 NOP2/Sun domain family, member 5

NUFIP1 nuclear fragile X mental retardation protein interacting protein 1

NVL nuclear VCP-like

NXT1 NTF2-like export factor 1

NXT2 nuclear transport factor 2-like export factor 2

PPAN peter pan homolog (Drosophila)

PA2G4 proliferation-associated 2G4, 38kDa

PAK1IP1 PAK1 interacting protein 1

PAPD5 PAP associated domain containing 5

PARN poly(A)-specific ribonuclease

PDCD11 programmed cell death 11

PELP1 proline, glutamate and leucine rich protein 1

PES1 pescadillo ribosomal biogenesis factor 1

PHAX phosphorylated adaptor for RNA export

PHF6 PHD finger protein 6

PIH1D1 PIH1 domain containing 1

PINX1 PIN2/TERF1 interacting, telomerase inhibitor 1

PNO1 partner of NOB1 homolog (S. cerevisiae)

PTRHD1 peptidyl-tRNA hydrolase domain containing 1

PWP1 PWP1 homolog (S. cerevisiae)

PWP2 PWP2 periodic tryptophan protein homolog (yeast)

RAI1 retinoic acid induced 1

RAN RAN, member RAS oncogene family

RBM19 RNA binding motif protein 19

RBM28 RNA binding motif protein 28

RCL1 RNA terminal phosphate cyclase-like 1

REXO1 REX1, RNA exonuclease 1 homolog (S. cerevisiae)

REXO2 REX2, RNA exonuclease 2 homolog (S. cerevisiae)

REXO4 REX4, RNA exonuclease 4 homolog (S. cerevisiae)

RIOK1 RIO kinase 1

RIOK2 RIO kinase 2

RNMTL1 RNA methyltransferase like 1

RPAP3 RNA polymerase II associated protein 3

RPF1 ribosome production factor 1 homolog (S. cerevisiae)

RPF2 ribosome production factor 2 homolog (S. cerevisiae)

RPS19BP1 ribosomal protein S19 binding protein 1

RRP1 ribosomal RNA processing 1 homolog (S. cerevisiae)

RRP12 ribosomal RNA processing 12 homolog (S. cerevisiae)

RRP36 ribosomal RNA processing 36 homolog (S. cerevisiae)

RRP7A ribosomal RNA processing 7 homolog A (S. cerevisiae)

RRP8 ribosomal RNA processing 8, methyltransferase, homolog (yeast)

RRP9 ribosomal RNA processing 9, small subunit (SSU) processome component, homolog (yeast)

RRS1 RRS1 ribosome biogenesis regulator homolog (S. cerevisiae)

RSAD1 radical S-adenosyl methionine domain containing 1

RUVBL1 RuvB-like 1 (E. coli)

RUVBL2 RuvB-like 2 (E. coli)

SBDS Shwachman-Bodian-Diamond syndrome

SDAD1 SDA1 domain containing 1

SHQ1 SHQ1, H/ACA ribonucleoprotein assembly factor

SKIV2L2 superkiller viralicidic activity 2-like 2 (S. cerevisiae)

SLFN14 schlafen family member 14

SPATA5 spermatogenesis associated 5

SPATA5L1 spermatogenesis associated 5-like 1

FTSJ3 FtsJ homolog 3 (E. coli)

TAF9 TAF9 RNA polymerase II, TATA box binding protein (TBP)-associated factor, 32kDa

TBL3 transducin (beta)-like 3

TCF25 transcription factor 25 (basic helix-loop-helix)

TCOF1 Treacher Collins-Franceschetti syndrome 1

TEX10 testis expressed 10

TFB1M transcription factor B1, mitochondrial

TGS1 trimethylguanosine synthase 1

TMA16 translation machinery associated 16 homolog (S. cerevisiae)

TMED5 transmembrane emp24 protein transport domain containing 5

TNPO1 transportin 1

TSR1 TSR1, 20S rRNA accumulation, homolog (S. cerevisiae)

TSR2 TSR2, 20S rRNA accumulation, homolog (S. cerevisiae)

TSR3 TSR3, 20S rRNA accumulation, homolog (S. cerevisiae)

TXNL4A thioredoxin-like 4A

UFD1L ubiquitin fusion degradation 1 like (yeast)

URB1 URB1 ribosome biogenesis 1 homolog (S. cerevisiae)

URB2 URB2 ribosome biogenesis 2 homolog (S. cerevisiae)

UTP11L UTP11-like, U3 small nucleolar ribonucleoprotein, (yeast)

UTP14A UTP14, U3 small nucleolar ribonucleoprotein, homolog A (yeast)

UTP15 UTP15, U3 small nucleolar ribonucleoprotein, homolog (S. cerevisiae)

UTP18 UTP18 small subunit (SSU) processome component homolog (yeast)

UTP20 UTP20, small subunit (SSU) processome component, homolog (yeast)

UTP23 UTP23, small subunit (SSU) processome component, homolog (yeast)

UTP3 UTP3, small subunit (SSU) processome component, homolog (S. cerevisiae)

UTP6 UTP6, small subunit (SSU) processome component, homolog (yeast)

VCP valosin containing protein

WDR12 WD repeat domain 12

WDR18 WD repeat domain 18

WDR36 WD repeat domain 36

WDR43 WD repeat domain 43

WDR46 WD repeat domain 46

WDR74 WD repeat domain 74

WDR75 WD repeat domain 75

WRAP53 WD repeat containing, antisense to TP53

XPO1 exportin 1 (CRM1 homolog, yeast)

XRN1 5'-3' exoribonuclease 1

XRN2 5'-3' exoribonuclease 2

ZCCHC4 zinc finger, CCHC domain containing 4

ZNHIT3 zinc finger, HIT-type containing 3

ZNHIT6 zinc finger, HIT-type containing 6

BMT2 base methyltransferase of 25S rRNA 2 homolog

BUD23 BUD23 rRNA methyltransferase and ribosome maturation factor

CCDC86 coiled-coil domain containing 86

DHX8 DEAH (Asp-Glu-Ala-His) box polypeptide 8

NOP53 glioma tumor suppressor candidate region gene 2

NOPCHAP1 chromosome 12 open reading frame 45

NLE1 notchless homolog 1 (Drosophila)

SLX9 family with sequence similarity 207, member A

NOL11 nucleolar protein 11

AQR aquarius homolog (mouse)

C1D C1D nuclear receptor corepressor

RSL1D1 ribosomal L1 domain containing 1

NIFK MKI67 (FHA domain) interacting nucleolar phosphoprotein

ZNF622 zinc finger protein 622

PDCD2 programmed cell death 2

PDCD2L programmed cell death 2-like

GEMIN5 gem (nuclear organelle) associated protein 5

RBM34 AT rich interactive domain 4B (RBP1-like) // RNA binding motif protein 34

BOP1 BOP1 ribosomal biogenesis factor

**Supplementary table S2.**

Characteristics of PAM50 and intrinsic breast cancer subtype cohorts from TCGA dataset

|  | PAM50 (n = 739) | Intrinsic breast  cancer subtype (n = 712) |
| --- | --- | --- |
| Age (years) |  |  |
| Mean (SD) | 58.1 (13.2) | 58.1 (13.3) |
| Median [Min: Max] | 58.0 [26.0 : 90.0] | 58.0 [26.0 : 90.0] |
| Gender |  |  |
| Female | 739 (100%) | 712 (100%) |
| Male | 0 (0%) | 0 (0%) |
| Tumour size |  |  |
| T1 | 195 (26.4%) | 186 (26.1%) |
| T2 | 408 (55.2%) | 421 (59.1%) |
| T3 | 71 (9.6%) | 77 (10.8%) |
| T4 | 24 (3.2%) | 25 (3.5%) |
| TX | 2 (0.3%) | 3 (0.4%) |
| Missing | 39 (5.3%) | 0 (0%) |
| Node invasion |  |  |
| N0 | 345 (46.7%) | 354 (49.7%) |
| N1 | 233 (31.5%) | 235 (33.0%) |
| Missing | 161 (21.8%) | 123 (17.3%) |
| Metastasis |  |  |
| M0 | 684 (92.6%) | 695 (97.6%) |
| M1 | 12 (1.6%) | 12 (1.7%) |
| Missing | 43 (5.8%) | 5 (0.7%) |
| Stage |  |  |
| Stage I | 112 (15.2%) | 109 (15.3%) |
| Stage II | 299 (40.5%) | 305 (42.8%) |
| Stage III | 103 (13.9%) | 100 (14.0%) |
| Stage IV | 4 (0.5%) | 4 (0.6%) |
| Missing | 221 (29.9%) | 194 (27.2%) |
| Histological type |  |  |
| Infiltrating Ductal Carcinoma | 611 (82.7%) | 580 (81.5%) |
| Infiltrating Lobular Carcinoma | 60 (8.1%) | 68 (9.6%) |
| Medullary Carcinoma | 4 (0.5%) | 3 (0.4%) |
| Mixed Histology | 28 (3.8%) | 23 (3.2%) |
| Mucinous Carcinoma | 5 (0.7%) | 5 (0.7%) |
| Other | 30 (4.1%) | 32 (4.5%) |
| Missing | 1 (0.1%) | 1 (0.1%) |
| Neoadjuvant treatment |  |  |
| Yes | 0 (0%) | 0 (0%) |
| No | 739 (100%) | 712 (100%) |

**Supplementary Table S3.**

Comparison of TCGA breast cancer sample classifications

| Intrinsic status^#^/PAM50^$^ | Luminal A  (n = 362) | Luminal B  (n = 172) | HER2  (n = 68) | TNBC  (n = 137) | NA  (n = 189) |
| --- | --- | --- | --- | --- | --- |
| Luminal A (n=486) | **289** | 123 | 5 | 19 | 50 |
| Luminal B (n=74) | 21 | **27** | 19 | 1 | 6 |
| HER2  (n=30) | 0 | 1 | **26** | 2 | 1 |
| TNBC  (n=122) | 3 | 1 | 10 | **101** | 7 |
| NA (=216) | 49 | 20 | 8 | 14 | **125** |

$: PAM50 classification relying on transcriptomics is directly given by the TCGA database

#: Intrinsic breast cancer status classification relying on detection of oestrogen, progesterone and HER2 receptors was determined using the positive or negative IHC status given by the TCGA database

**Supplementary table S4.**

Antibodies used in Western blot

| **Primary antibodies** | | |  | |  | |  | |
| --- | --- | --- | --- | --- | --- | --- | --- | --- |
| **Protein** | **M.W.** | **Dilution** | | **Provider / reference** | | **RRID** | |  |
| PARP | 120 | 1/1000 | | Cell Signalling - #9532S | | AB_659884 | |  |
| PARP 1 total | 133 | 1/1000 | | Abcam - ab137653 | | n/a | |  |
| Cleaved PARP | 89 | 1/1000 | | Cell Signalling - #5625 | | AB_10699459 | |  |
| Caspase 3 | 35 | 1/100 | | Enzo - ALX-804-305-C100 | | AB_2050938 | |  |
| Cleaved caspase 3 | 17 | 1/1000 | | Cell Signalling - #9661 | | AB_2341188 | |  |
| Fibrillarin | 37 | 1/1000 | | Abcam - Ab166630 | | AB_2928100 | |  |
| Ku80 | 80 | 1/2000 | | Abcam - Ab119935 | | AB_10899161 | |  |
| β-Actin | 42 | 1/5000 | | Sigma - A5441 | | AB_476744 | |  |
| Histone H3 | 17 | 1/5000 | | Abcam - ab1791 | | AB_302613 | |  |
| γH2A.X | 17 | 1/1000 | | Millipore - 05-636 | | AB_309864 | |  |
| Phospho-Histone H3 (ser10) | 17 | 1/1000 | | Cell Signalling - #9701 | | AB_331535 | |  |
|  |  |  | |  | |  | |  |
| **Secondary antibodies** | | |  | |  | |  | |
| **Protein** |  | **Dilution** | | **Provider / reference** | | **RRID** | |  |
|  |  |  | |  | |  | |  |
| Anti-rabbit HRP |  | 1/5000 | | Cell Signalling - #7074 | | AB_2099233 | |  |
| Anti-mouse HRP |  | 1/5000 | | Cell Signalling - #7076 | | AB_330924 | |  |
| Anti-rabbit StarBright 700 |  | 1/5000 | | BioRad - 12004161 | | AB_2721073 | |  |
| Anti-mouse StarBright 520 |  | 1/5000 | | BioRad - 12005867 | | n/a | |  |
|  |  |  | |  | |  | |  |

n/a = non applicable

RRID = Research Resource Identifiers (https://rrid.site)

**Supplementary table S5.**

Antibodies used for immunofluorescence

| **Protein** | **Dilution** | | **Provider / reference** | | **RRID** |
| --- | --- | --- | --- | --- | --- |
| FBL | 1/4000 | Abcam - Ab5821 | | AB_2105785 | |
| Phospho-H3(S10) | 1/1000 | Cell Signalling - 9701S | | AB_331535 | |
|  | 1/1000 | Merck - 06-570 | | AB_310177 | |
| Gamma H2Ax | 1/6000 | Merck - 05-636 | | AB_309864 | |
| 53BP1 | 1/500 | Cell Signalling - 4937-S | | AB_10694558 | |
| Alexa-Fluor 488 anti-Rabbit | 1/1000 | Invitrogen - A11008 | | AB_143165 | |
| Alexa-Fluor 488 anti-Mouse | 1/1000 | Invitrogen - A11001 | | AB_2534069 | |
| Alexa-Fluor 555 anti-Rabbit | 1/1000 | Invitrogen - A21428 | | AB_141784 | |
| Alexa-Fluor 555 anti-Mouse | 1/1000 | Invitrogen - A21424 | | AB_141780 | |
| Alexa-Fluor 647 anti-Rabbit | 1/1000 | Invitrogen - A21245 | | AB_2535813 | |
| Alexa-Fluor 647 anti-Mouse | 1/1000 | Invitrogen - A21235 | | AB_2535804 | |
