# Supplementary material for: Fibrillarin-mediated ribosomal RNA maturation is a novel therapeutic vulnerability in triple-negative breast cancer
Source: Breast Cancer Res. 2025 Nov 13;27:202. doi: 10.1186/s13058-025-02163-x (PMC12616997; doi:10.1186/s13058-025-02163-x)

Figure 2B

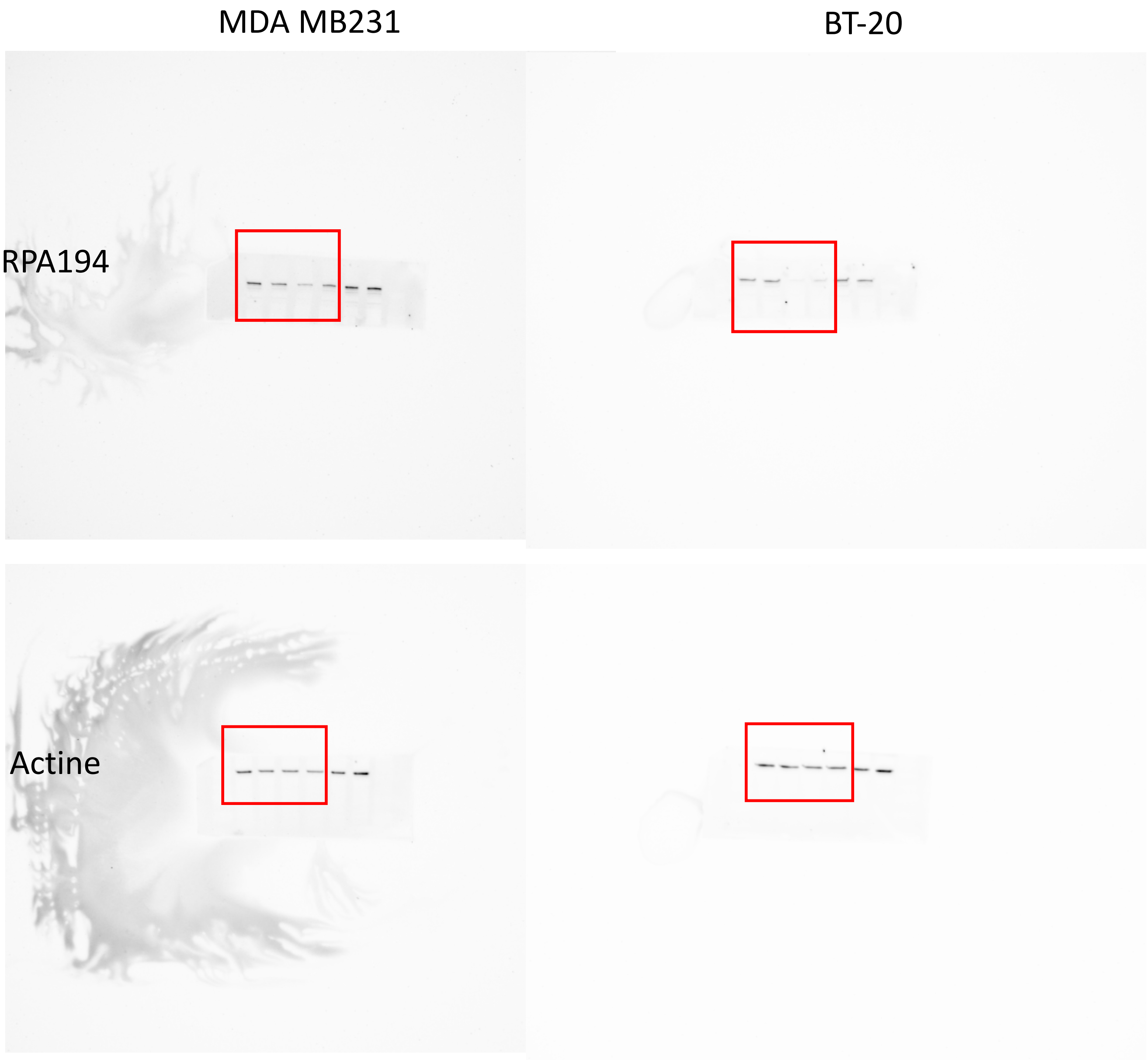

Figure 2D

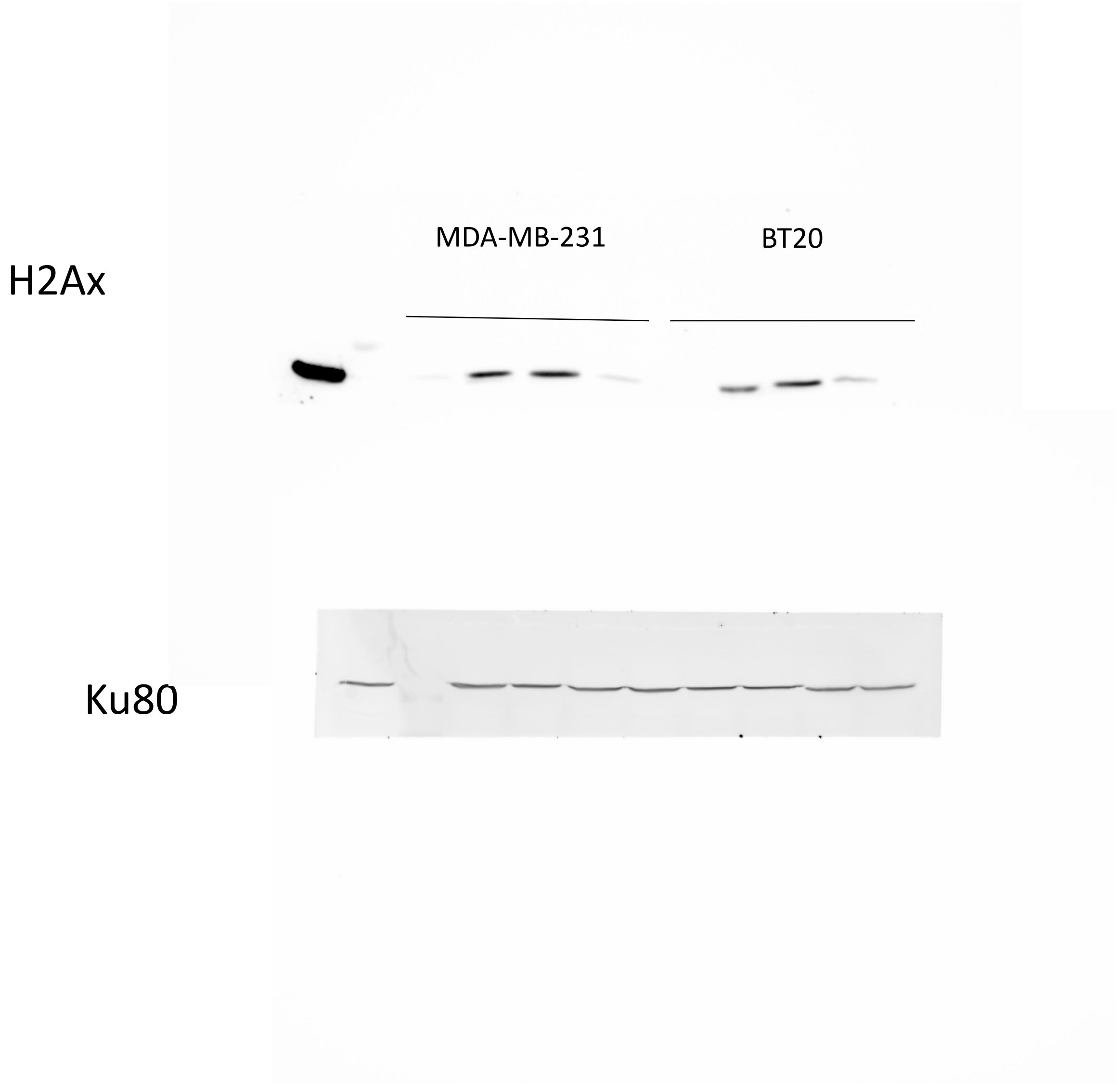

Figure 5c

MDA MB231

BT-20

FBL

H3

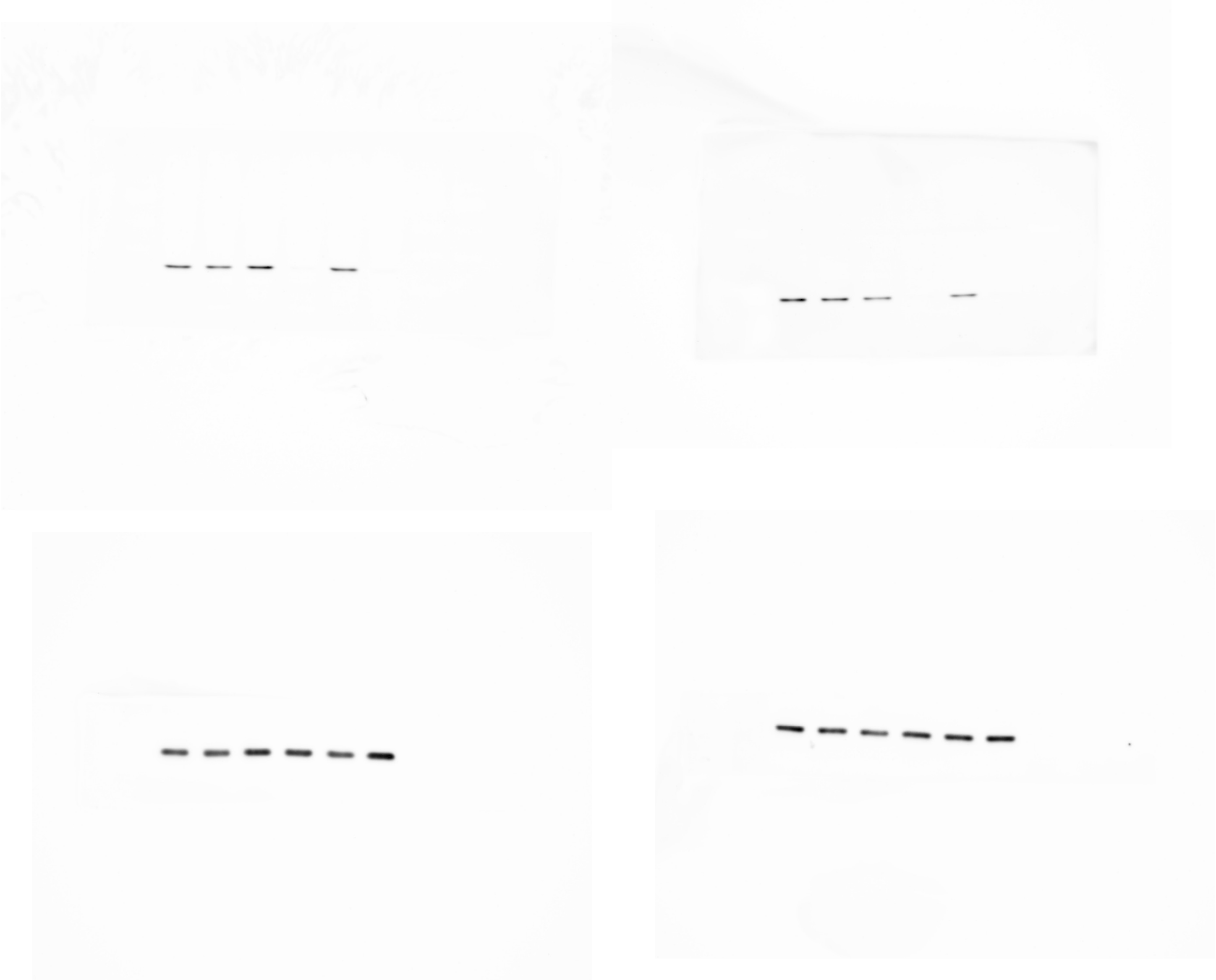

Figure 5E

MDA MB231

ITS 1

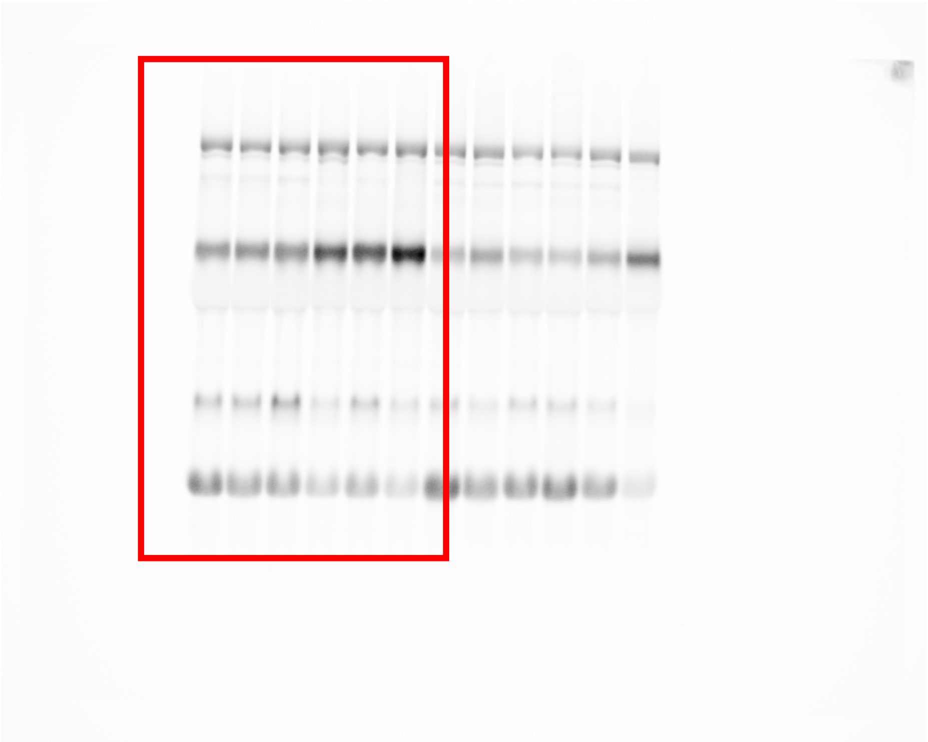

ITS 2

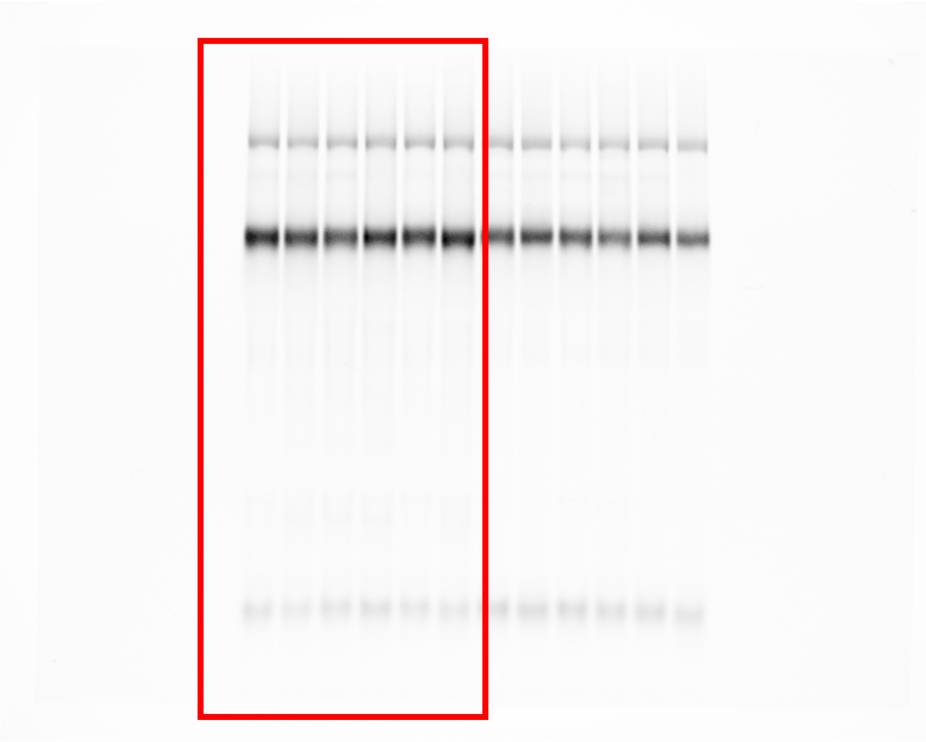

Figure 5E

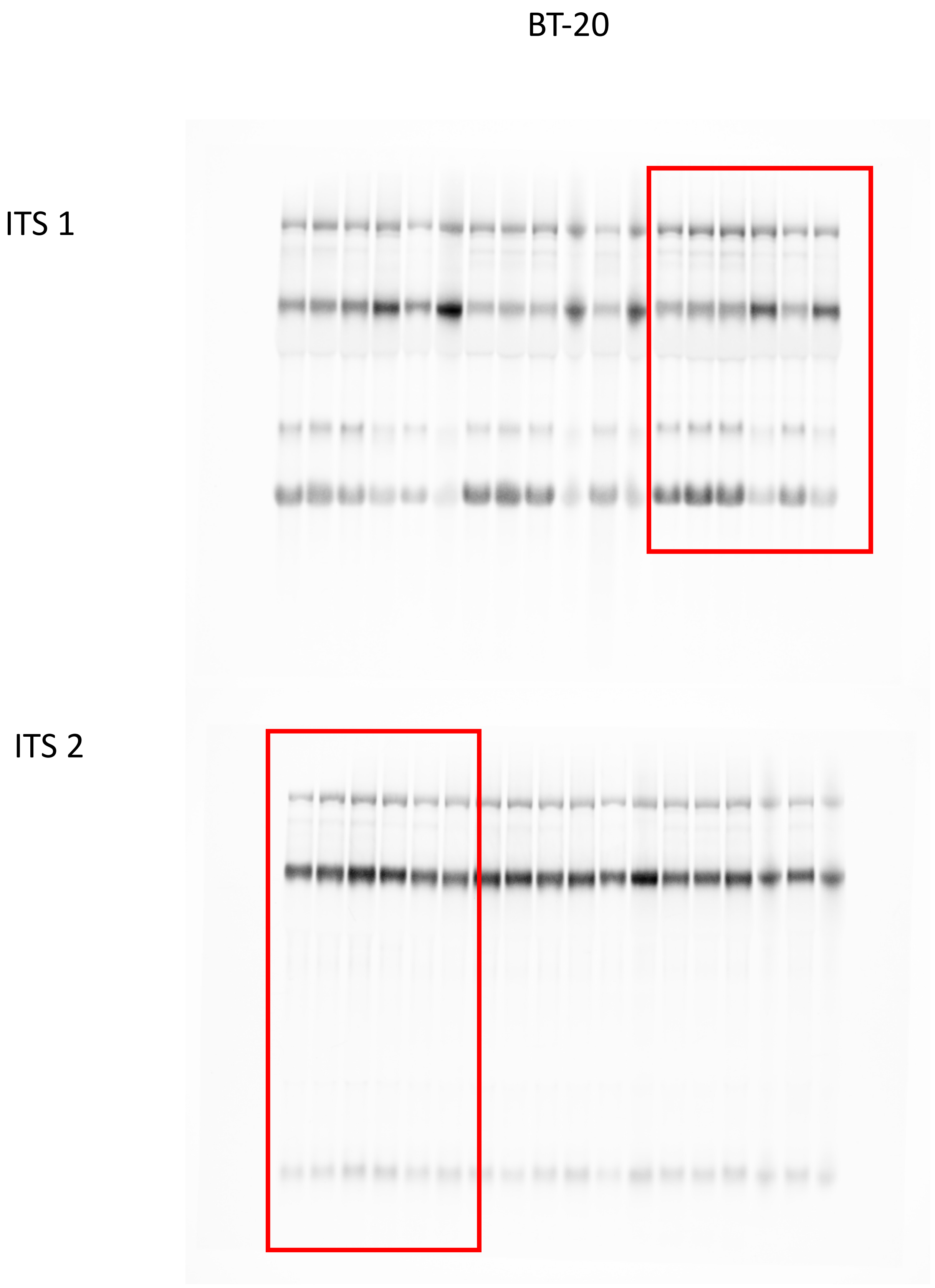

Figure 5F

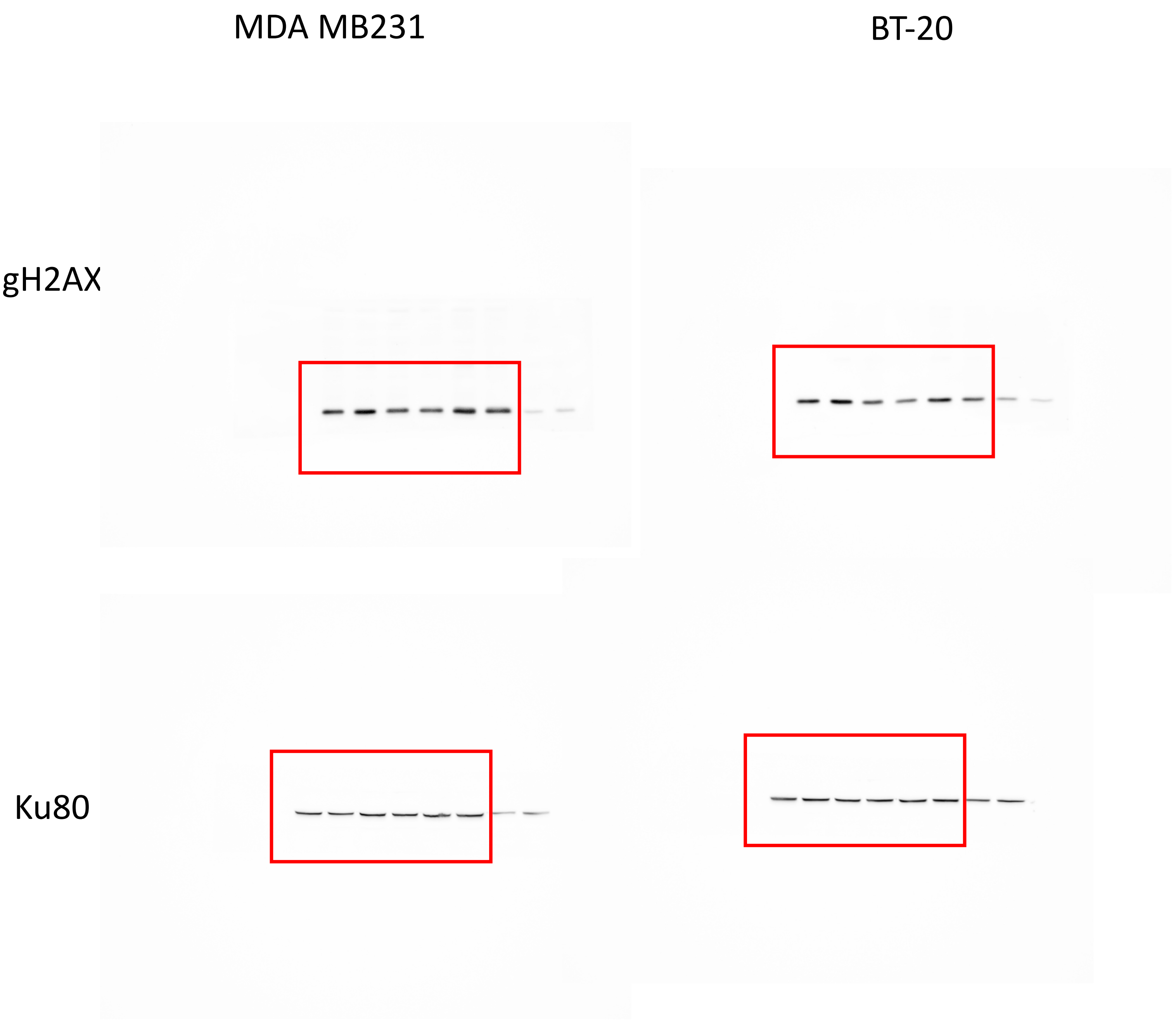

Figure7B

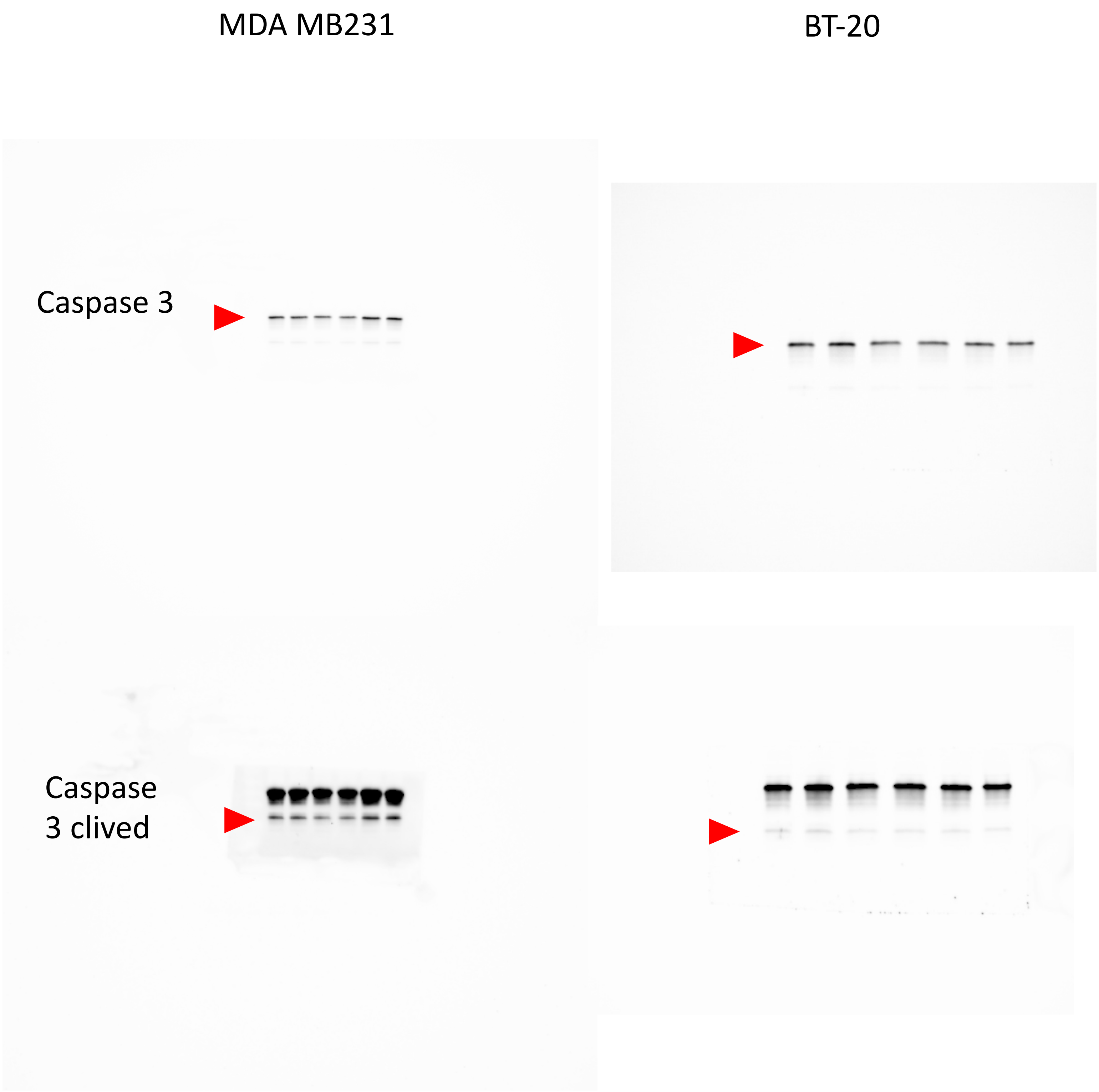

Figure sup3B

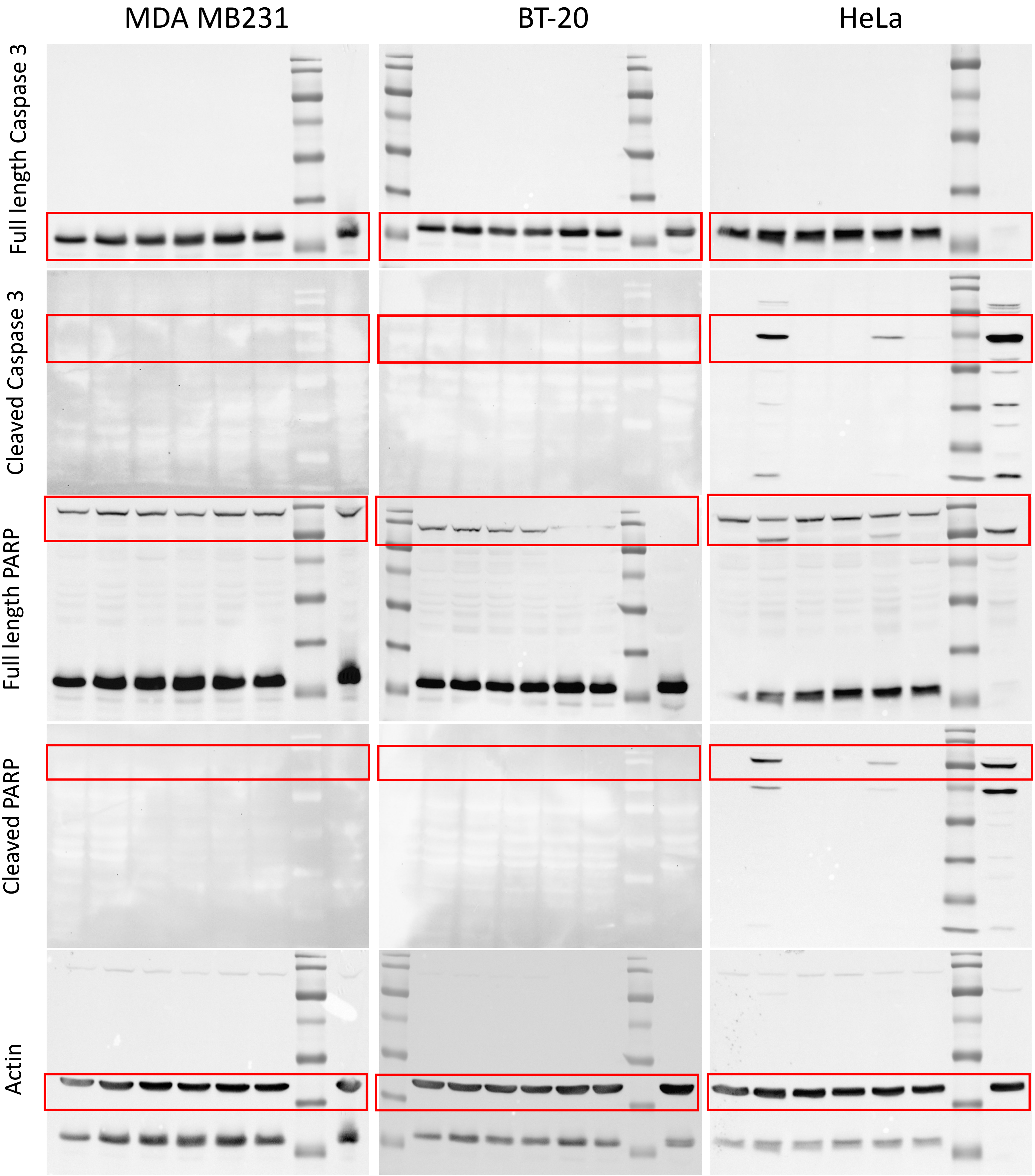

Figure sup3D

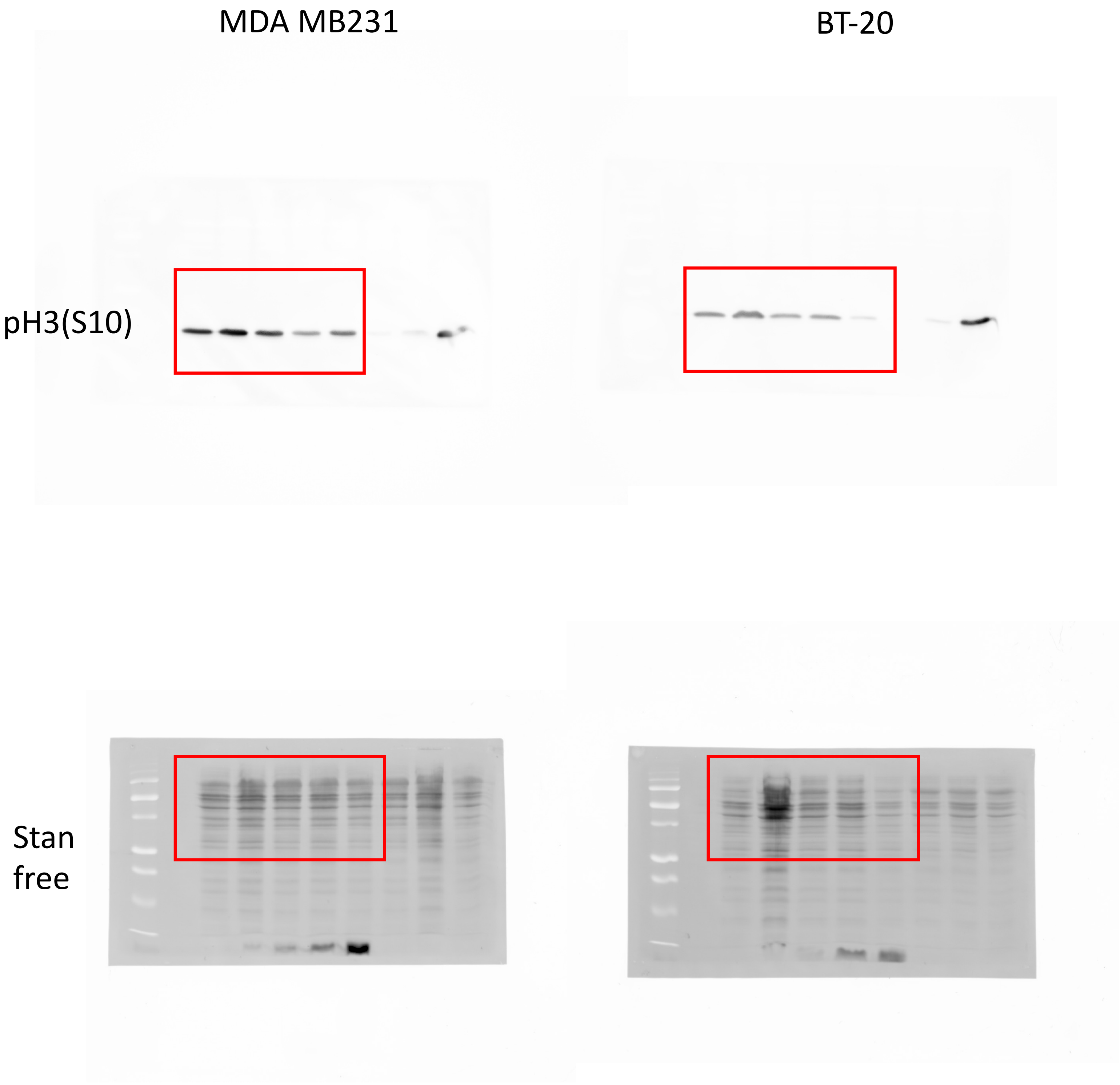

Figure sup3D

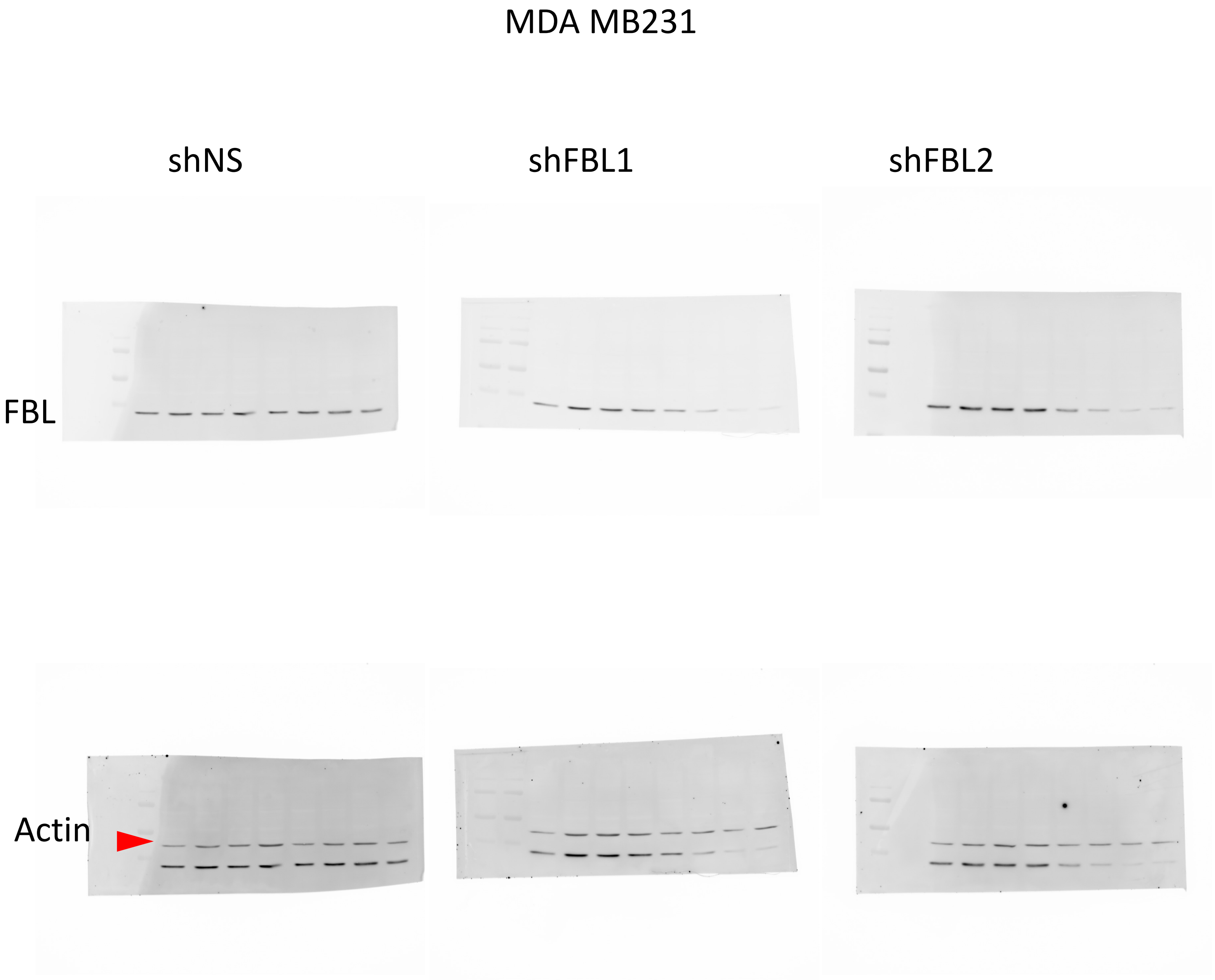

Figure sup3D

BT-20

shNS shFBL1 shFBL2

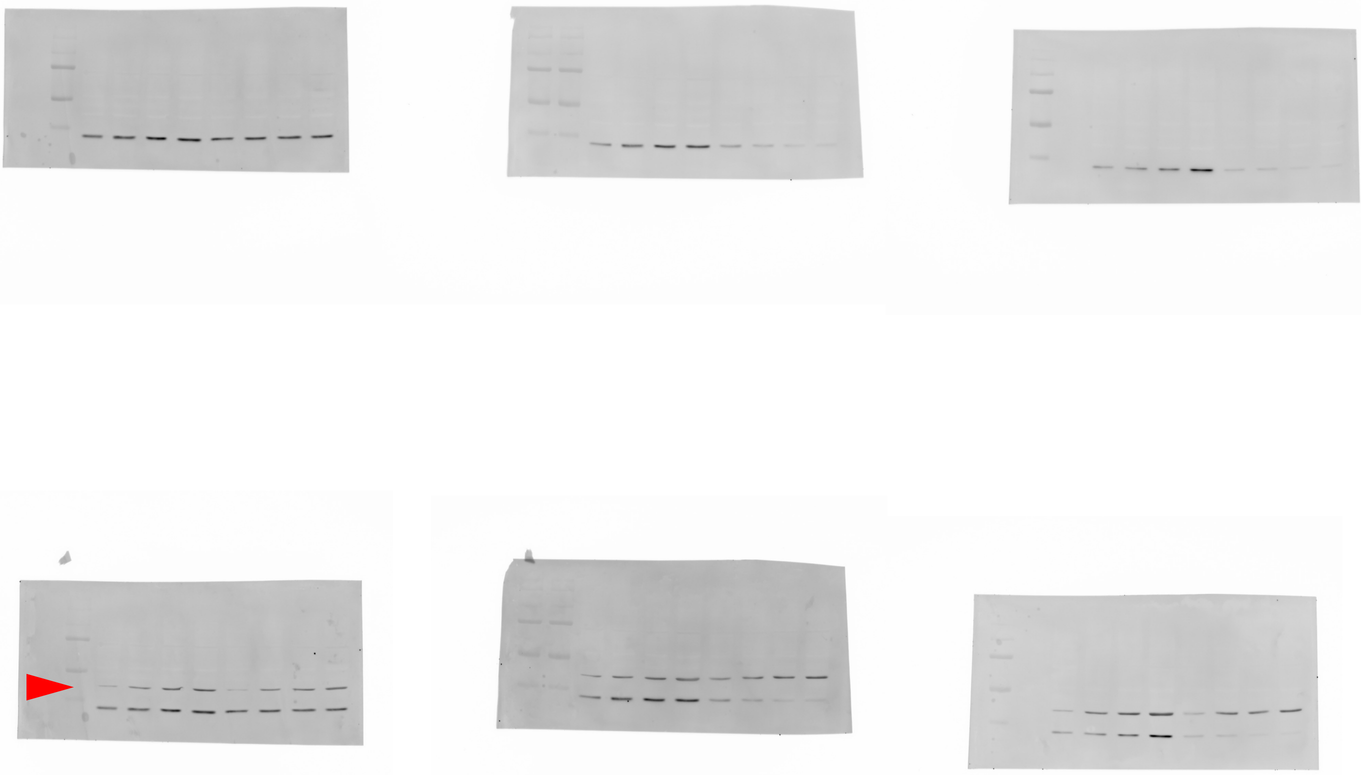

FigureSup6A

MDA MB231

BT-20

Caspase 3

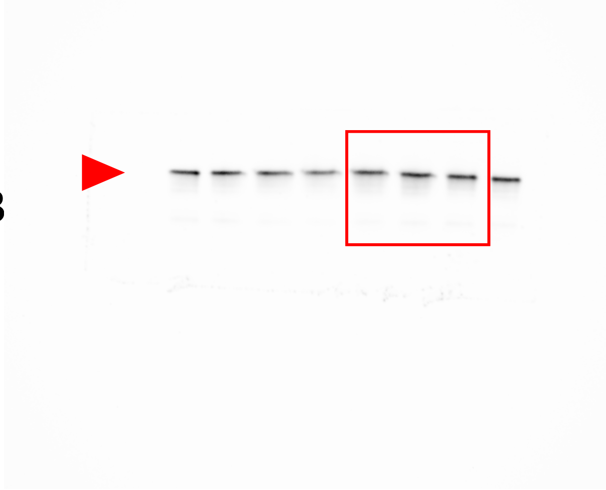

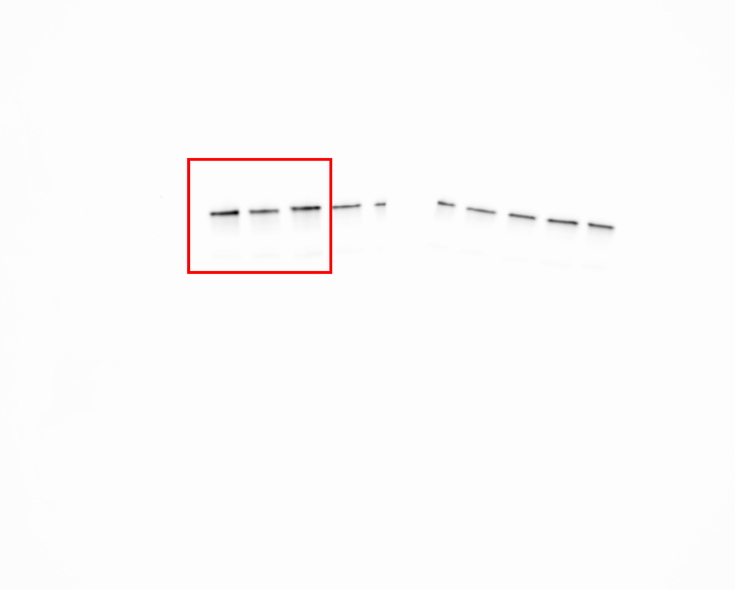

Caspase
3 clived

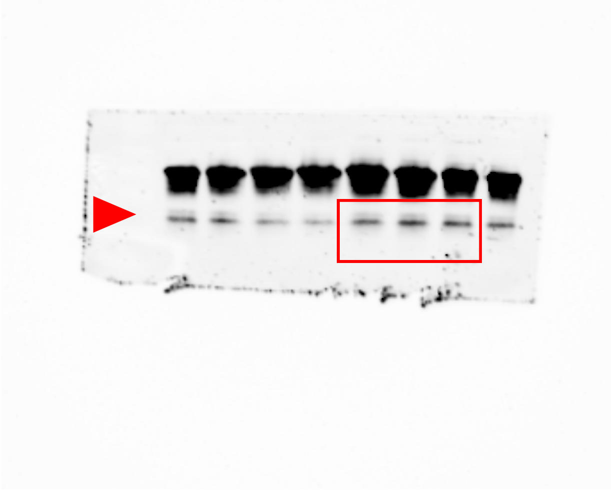

FigureSup6A

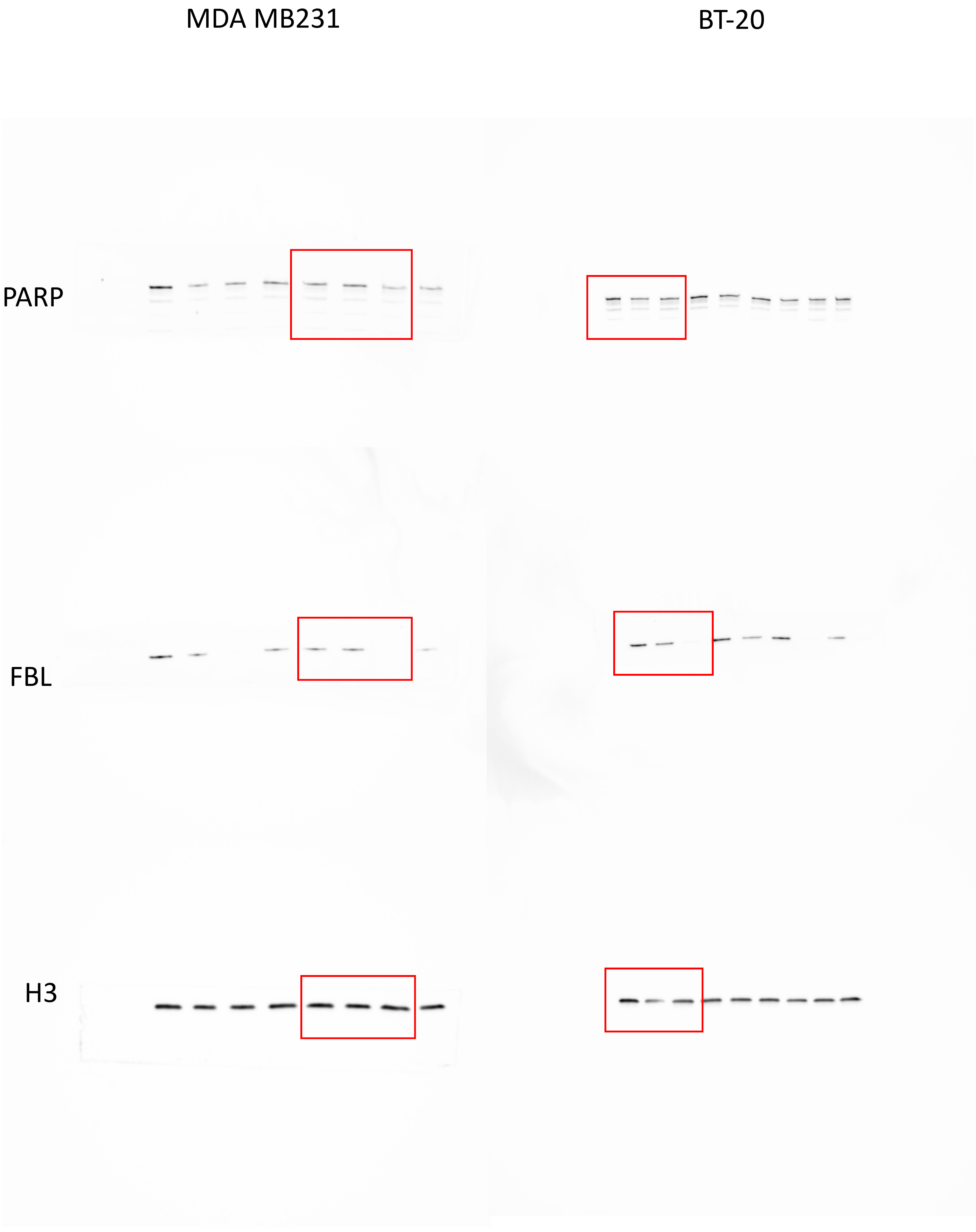

FigureS7B

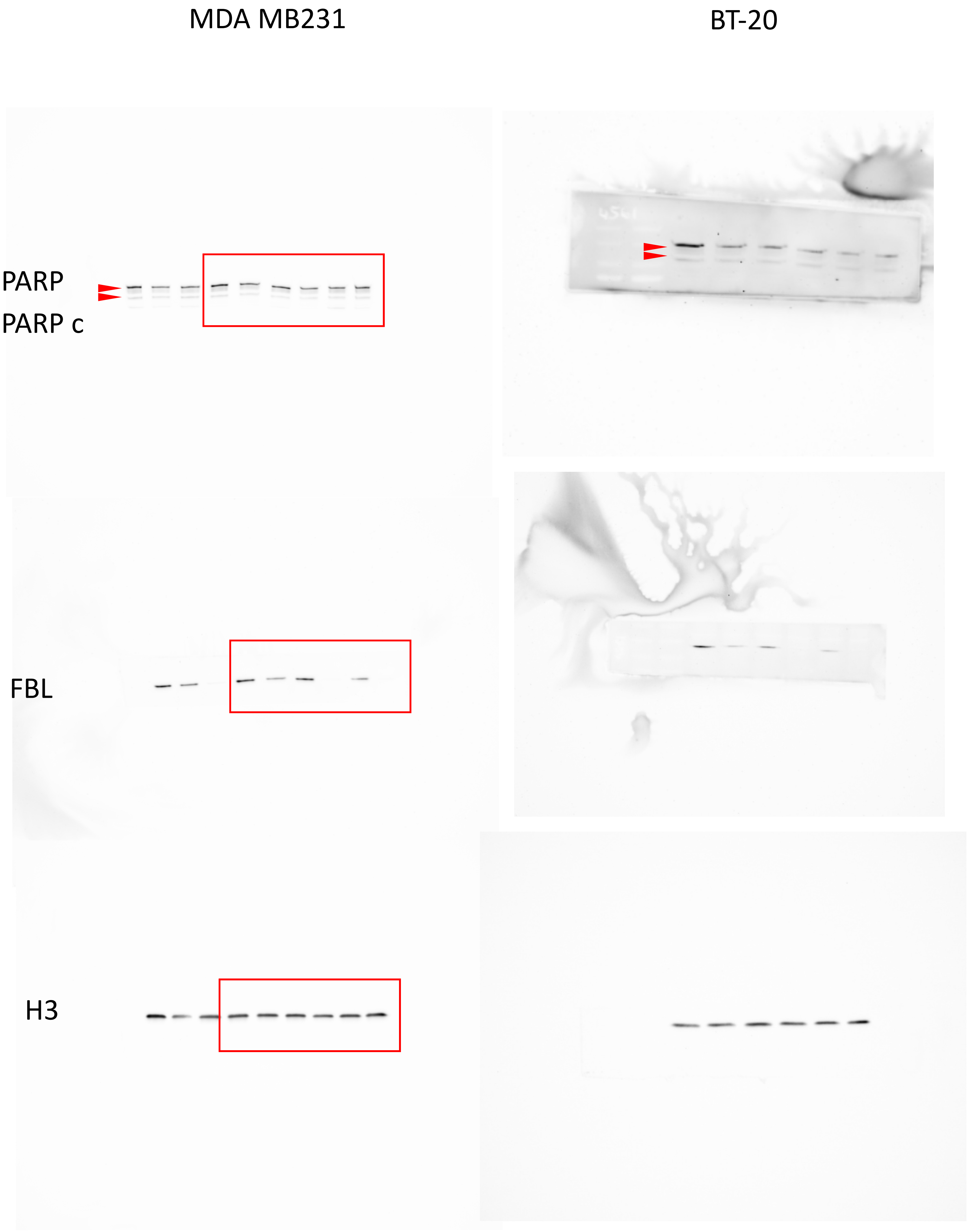

Supplement: Supplementary file 5 — Supplementary Material 5 [file 13058_2025_2163_MOESM5_ESM.pdf]
